# Variable High-Frequency Deep Brain Stimulation of the Subthalamic Nucleus for Speech Disorders in Parkinson's Disease: A Case Report

**DOI:** 10.3389/fneur.2019.00379

**Published:** 2019-04-16

**Authors:** Chencheng Zhang, Yixin Pan, Haiyan Zhou, Qing Xie, Bomin Sun, Chuanxin M. Niu, Dianyou Li

**Affiliations:** ^1^Department of Functional Neurosurgery, School of Medicine, Ruijin Hospital, Shanghai Jiao Tong University, Shanghai, China; ^2^Department of Neurology, School of Medicine, Ruijin Hospital, Shanghai Jiao Tong University, Shanghai, China; ^3^Department of Rehabilitation Medicine, School of Medicine, Ruijin Hospital, Shanghai Jiao Tong University, Shanghai, China; ^4^Med-X Research Institute, School of Biomedical Engineering, Shanghai Jiao Tong University, Shanghai, China

**Keywords:** variable high frequency, subthalamic nucleus, deep brain stimulation, dysarthria, Parkinson's disease

## Abstract

**Background and Importance:** It is known that subthalamic nucleus deep brain stimulation (STN-DBS) at a fixed high frequency (>100 Hz) improves the primary motor symptoms of Parkinson disease (PD), but this stimulation does not improve or may even exacerbate the later-occurring axial symptoms and signs in PD (e.g., problems with gait or speech). Recent evidence suggests that STN-DBS at a fixed lower frequency (< 100 Hz) can improve speech and gait, but may worsen the tremor in PD.

**Clinical Presentation:** The case involved a female patient who developed severe speech problems after 16 years high-frequency STN-DBS for PD. The tremor and dysarthria symptoms were both effectively treated by applying variable-frequency stimulation (VFS) containing only a combination of high frequencies.

**Conclusion:** VFS containing several higher frequencies improved both the tremor and axial signs including speech problems in our patient. This case report suggests that VFS may be of clinical utility in the management of advanced PD, but this should be further verified in larger well-controlled studies.

## Background and Importance

High-frequency deep brain stimulation of the subthalamic nucleus deep brain stimulation (STN-DBS) improves the primary motor symptoms of Parkinson's disease (PD). However, this stimulation at a fixed high frequency does not improve or may even exacerbate the axial symptoms and signs (such as problems with gait, speech, or swallowing) that often emerge over the long-term course of treatment and disease (1). Recent evidence suggests that STN-DBS at a fixed lower frequency (< 100 Hz) could improve speech and gait (2, 3). However, the tremor might worsen significantly with fixed low-frequency stimulation (4). Here, we present a case of PD that was treated effectively by applying variable-frequency stimulation (VFS) containing only a combination of high frequencies. A written informed consent was obtained from the patient, both for participation and for the academic publication of this case report.

## Clinical Presentation

**Figure 1:**
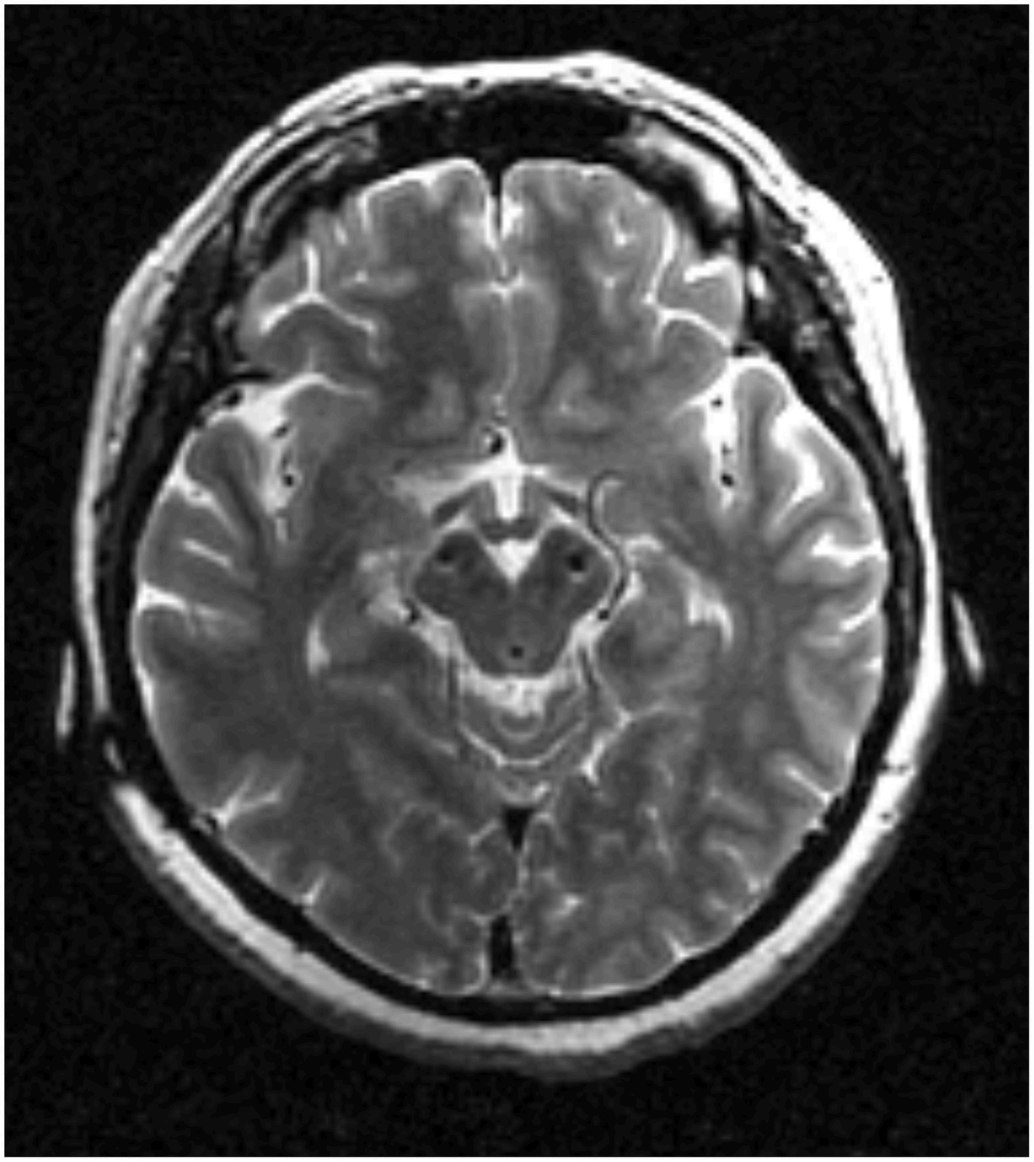
The most ventral DBS contacts location.

The case involved a female patient who developed severe speech problems after long-term high-frequency STN-DBS for PD. In 1998, at the age of 23, she was diagnosed with PD. In 2002, she received bilateral STN-DBS (Kinetra 7428, Medtronic, Minneapolis, MN, USA) for severe medication-resistant tremor. The position of the most ventral DBS contacts was shown in Figure 1. After STN-DBS onset, she first decided to reduce the dosage of anti-Parkinson medications and then stopped taking the drugs altogether. One year after surgery, she was medication-free and gave birth to a baby. She received subsequent battery replacements in 2006, 2009, and 2012. For over a decade, her motor symptoms responded well to DBS at 170 Hz. However, from April 2015 onwards, she experienced increasing difficulties in standing up from a sitting position and with her speech/phonation. The adjustments made to her DBS parameters and medication were not helpful. Post-operative magnetic resonance imaging confirmed that the DBS leads were correctly located in the STN and had not migrated. In December 2015, we replaced the DBS battery of the patient with a rechargeable battery (G102, PINS, Beijing, China). As stimulation at a fixed low frequency might exacerbate the tremor evident in this patient, we explored the value of VFS in treating her motor symptoms, which was made possible by the battery change (5). The same parameters were selected: right, 1-2-3-Case+, 3.55 V, 100 us, 170 Hz; left, 6-7-C+, 3.35 V, 90 us, 170 Hz.

We evaluated the effects of three different sets of VFS on the patient's motor function. Initially, the frequencies used in each set were randomly chosen from a group of six frequencies (170, 160, 145, 125, 90, 60 Hz). Ten minutes after the delivery of each VFS stimulus, a speech therapist assessed the patient's vocal and speech performance by taking into account (a) the quality of articulation when she pronounced her name, date of birth, and an 8-syllable Chinese tongue-twister; (b) the maximum phonation time when pronouncing /ah/; and (c) the loudness of the sound of her voice, as indicated by the maximum sound pressure level while the patient produced a loud, clear sound for as long as possible (UT-352, Uni-Trend Technology, Ltd., Shenzhen, China). A movement disorder specialist also performed follow-up motor assessments using established clinical instruments. Other stimulation parameters (i.e., the contacts, amplitude, pulse width) were kept the same while frequencies were varied across VFS sets.

The first set of VFS parameters used contained two low-frequency components (90 and 60 Hz). Following the application of this set, the patient could no longer speak, and her tremor immediately recurred. We therefore excluded these low frequencies from further consideration. Next, we evaluated our second set, involving 160 Hz (10 s), 145 Hz (15 s), 125 Hz (10 s), 145 Hz (15 s), and 160 Hz (10 s). Note that 145 and 160 Hz were used twice within this set as a 1-min loop. The second VFS set was found to alleviate her bradykinesia, muscle rigidity, and axial symptoms on day one and 1 month follow-ups (Table 1). We then evaluated our third set involving 160, 155, 145, 130, and 125 Hz (10 s) at the 1 month follow-up. Similar to the second parameter set, the third VFS set improved the patient's bradykinesia, rigidity, and axial symptoms at the 3 months follow-up, as compared to no STN-DBS treatment or fixed high-frequency STN-DBS (Table 1). Thus, the two VFS sets were both effective for primary motor symptoms, but the third set improved the axial symptoms better than the second set.

**Table 1:** Patient's motor symptom severity before and after variable-frequency stimulation.^*^

**Clinical variable**	**Off**	**HFS**	**2nd VFS (1-day follow-up)**	**2nd VFS (1-month follow-up)**	**3rd VFS (3-months follow-up)**
Total	90	50	45	39	35
Tremor	10	6	6	5	5
Rigidity	20	8	8	2	2
Bradykinesia	40	24	22	22	22
Axial symptoms	20	12	9	10	6
Speech	4	3	3	2	2
Gait	4	2	2	2	1
Posture	4	2	1	2	1
Postural stability	4	2	2	2	2
Arise from chair	4	3	1	2	0
TUG (sec)	Unable to complete	10	12	10	11
Hoehn-Yahr Stage	5	3	3	3	3
GFQ	NA	36	32	32	32
Voice loudness	NA	Max duration = 2.2, max SPL = 89.0	Max duration = 3.3, max SPL = 98.1	Max duration = 2.3, max SPL = 93.2	Max duration = 3.4, max SPL = 97.6

* *Motor symptom severity was assessed by using the Unified Parkinson's Disease Rating Scale (UPDRS)-III, unless indicated otherwise. Off, no STN-DBS; HFS, 170 Hz; HFS, High Frequency Stimulation; LFS, Low Frequency Stimulation; TUG, Time Up and Go test; GFQ, Gait and Falls Questionnaire; SPL, Sound Pressure Level; NA, Not Available*.

## Discussion and Conclusion

In PD, axial symptoms, and signs involving problems with gait and speech are common, especially in advanced stages of the disease. For the affected, these symptoms often lead to functional impairment and a lower perceived quality of life. It is known that STN-DBS treatment using fixed high frequencies, while being effective in controlling the primary motor symptoms of PD, may induce or aggravate speech and voice dysfunctions (1). VFS containing low frequencies has been reported to relieve severe subthalamic stimulation-induced dysarthria, yet this stimulation has also been found to worsen the tremor (2). Our results confirm the latter observation by showing that VFS containing low frequencies alone worsened the tremor in the present case. By contrast, VFS containing several higher frequencies improved both the tremor and axial signs including speech problems in our patient.

This case report describes the application of new paradigms for DBS programming, made possible by technological advances. Unfortunately, for the different sets of VFS tested here, the stimulation amplitude and pulse width could not be varied. One possible explanation for the scarce efficacy of the first VFS set (containing high and low frequencies) could be that low frequencies typically require a higher stimulation intensity to be at least as effective as the high frequencies. The differences between the two high-frequency sets of VFS implied that switching more frequently seems to be advantageous. Although assessment is usually done 30 min after stimulation, we assessed her speech after 10 min because this patient is very sensitive and reached a stable clinical effect quickly. Furthermore, the data is comparable as the conditioning followed the same protocol.

These observations indicate that VFS may be of clinical utility in the management of advanced PD. Future large-scale studies are needed to confirm our findings and elucidate the mechanism of VFS, and to establish whether it alleviates the detrimental effect of HFS, DBS or has a beneficial effect.

## Ethics Statement

This study was approved by the ethics committee of Ruijin Hospital, Shanghai Jiaotong University. The patient gave her consent for participation and anonymity publication.

## Author Contributions

CZ, BS, DL, and CN designed this study. QX and CN performed the speech assessment. YP, DL, and HZ conducted the DBS programing and motor assessment. CZ, DL, and CN wrote this paper with input from all co-authors.

### Conflict of Interest Statement

CZ and DL received honoraria and travel expenses from Medtronic Inc. (Minneapolis, MN, USA), PINS Medical Co., Ltd. (Beijing, China), and SceneRay Corp., Ltd. (Suzhou, China). BS received research support from PINS Medical Co., Ltd. and SceneRay Corp., Ltd. (donated devices). The remaining authors declare that the research was conducted in the absence of any commercial or financial relationships that could be construed as a potential conflict of interest.
